# Discrepancy between international normalized ratio measurements in a heterozygous carrier of *F10*:p.Gly244Arg with recurrent venous thromboembolism: a case report

**DOI:** 10.1016/j.rpth.2025.103298

**Published:** 2025-12-09

**Authors:** Nikolaj Julian Skrøder Nytofte, Emil List Larsen

**Affiliations:** Department of Internal Medicine, Centre for Thrombosis and Anticoagulation, Næstved, Slagelse, Ringsted (NSR) Hospitals, Næstved, Denmark

**Keywords:** case reports, factor X, international normalized ratio, prothrombin time, venous thromboembolism

## Abstract

**Background:**

The international normalized ratio (INR) is designed to monitor vitamin K antagonist (VKA) treatment. Before patients start a self-managing VKA program, parallel measurements are conducted to compare point-of-care testing (POCT) INR with venous INR samples. Previously, genetic variants in *F7* have shown discrepancies in INR measurements when thromboplastins from different species were used. It is unknown whether genetic variants in *F10* affect INR measured with thromboplastins from different species.

**Key Clinical Question:**

Does *F10*:p.Gly244Arg heterozygosity affects the INR when measured using rabbit compared with human thromboplastin?

**Clinical Approach:**

A patient self-managing warfarin treatment had a recurrent venous thromboembolism during VKA treatment. The POCT therapeutic range was low (ie, 1.6-2.4) based on parallel measurements of POCT INR (human thromboplastin) and venous INR (rabbit thromboplastin). Subsequently, it was noted that the patient had a spontaneous increase in INR (1.3), and the patient was found to be a heterozygous carrier of *F10*:p.Gly244Arg.

**Conclusion:**

Genetic variants in *F10* may also interfere with INR or prothrombin time measurements when different thromboplastins are used. This case illustrates that discrepancies in INR measurements with different thromboplastins should prompt consideration of genetic variants in *F10* and *F7* to ensure sufficient anticoagulant VKA treatment.

## Essentials

• Warfarin treatment can be monitored by a point-of-care INR test.
• Discrepancies in INR measurements have previously been observed in carriers of genetic variants in *F7.*
• Here, a heterozygote carrier of *F10*:p.Gly244Arg had different INR values depending on the thromboplastin used.
• Genetic variants in *F10* should also be considered when the INR result depends on the thromboplastin used.

## 1 Introduction

Coagulation factor (F)X is a vitamin K-dependent protein synthesized in the liver. FX is activated either through the intrinsic or extrinsic pathway and is the most important activator of prothrombin [1]. FX deficiency results in prolonged prothrombin time (PT) and activated partial thromboplastin time. Suspicion of inherited FX deficiency is supported by an immunological, clotting, or chromogenic assay measuring FX antigen or activity, and confirmed by genetic testing. Severe FX deficiency is rare and caused by homozygosity or compound heterozygosity of disease-causing genetic variants in *F10*, whereas heterozygous carriers are usually asymptomatic with no or minor bleeding symptoms [1].

## 2 Case Report

A 68-year-old male presented with complaints of a swollen and painful left leg. Doppler ultrasonography confirmed deep venous thrombosis (DVT) of the left popliteal and proximal crural veins. The patient underwent laser ablation and resection of varices 1 week before the onset of symptoms. Medical history included a spontaneous increase in the international normalized ratio (INR) diagnosed 11 years earlier (INR = 1.3), measured in a venous blood sample, and an unprovoked pulmonary embolism diagnosed 6 years earlier. The patient had been treated with warfarin since the diagnosis of pulmonary embolism and was followed in a patient self-management program. Before starting patient self-management, point-of-care test (POCT) INR (CoaguChek INRange, Roche Diagnostics) and laboratory plasma INR (STA-R Max, Triolab A/S) results were compared (Figure). The POCT showed a significantly lower INR than the laboratory plasma results, and, as such, a therapeutic range of 1.6 to 2.4 for POCT INR was chosen to obtain laboratory plasma results between 2.0 and 3.0. Prior to the development of DVT, the time in the therapeutic range was 87%. Warfarin treatment was changed to dalteparin 200 international unit (IU)/kg once daily for 30 days followed by rivaroxaban 20 mg once daily Subsequent tests showed an activated partial thromboplastin time of 33 seconds (reference interval: 27-40 seconds), plasma FII of 0.81 × 10^3^ IU/L (reference interval: 0.70-1.46 × 10^3^ IU/L), plasma FVII of 0.71 × 10^3^ IU/L (reference interval: 0.67-1.43 × 10^3^ IU/L), and plasma FX of 0.47 × 10^3^ IU/L (reference interval: 0.7-1.52 × 10^3^ IU/L). Whole-genome sequencing revealed that the patient was a heterozygous carrier of the pathogenic missense variant *F10*:c.730G>A,p.(Gly244Arg). The patient provided oral and written informed consent for presenting and puclication of the case-report.

**Figure:**
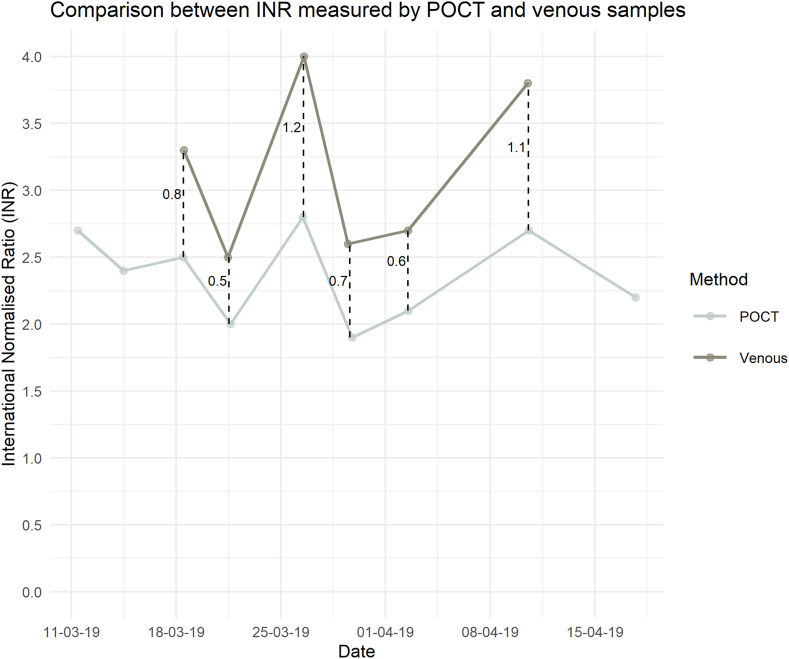
Parallel measurements of the international normalized ratio (INR) between point-of-care test (POCT) INR (CoaguChek INRange) and laboratory venous INR samples for the reported case, who was a heterozygous carrier of *F10*:p.Gly244Arg. The x-axis represents dates (dd-mm-yy). The dashed vertical lines with numbers indicate the differences between the POCT and venous INR measurements. The graph was created using R (R Foundation for Statistical Computing).

## 3 Discussion

Here, we report a case of recurrent venous thromboembolism provoked by minor surgery in a patient treated with warfarin. The patient continued anticoagulation treatment during the periprocedural period. We noted that the laboratory tests of INR measurements in venous blood samples increased spontaneously before anticoagulant treatment was initiated. Plasma FII and FVII were normal, but a clotting assay using FX-depleted plasma showed decreased plasma FX activity. Genetic analysis revealed heterozygosity for *F10*:p.(Gly244Arg), which is a known pathogenic variant resulting in severe type 1 FX deficiency in homozygous and compound heterozygous patients [[2], [3], [4], [5]]. The patient was self-managing warfarin treatment using a POCT (CoaguChek INRange), and the quality of anticoagulation was excellent. However, the therapeutic range using CoaguChek INRange was low (1.6-2.4), based on several tests comparing INR on the point-of-care device with laboratory INR from venous blood sample values in the therapeutic range (2.0-3.0; Figure). Laboratory INR was analyzed using rabbit thromboplastin, while CoaguChek INRange INR was analyzed using human thromboplastin. Interestingly, discrepancies in PT levels between rabbit and human thromboplastin have been noted in some FVII variants, resulting in prolonged PT with rabbit thromboplastin compared with human thromboplastin [6]. As with FVII, FX binds to phospholipids [2], thus providing a putative mechanism for the observed differences in PT using different thromboplastins. Previously, one homozygous *F10*:p.(Gly244Arg) carrier actually showed different PTs across different assays. However, this was assumed to be caused by pharmacological treatment prior to blood sampling [5]. Decreased interaction between *FX*:p.Gly244Arg in rabbit thromboplastin, resulting in decreased thrombin generation *in vitro* compared with PT analyzed with human thromboplastin, would explain these findings. Furthermore, this putative *in vitro* phenomenon would hamper the use of INR analysis with rabbit thromboplastin in this patient, as it would overestimate the INR. The falsely increased INR could have resulted in a dangerous situation, where the patient was not sufficiently anticoagulated, which, in part, could explain the recurrent DVT despite ongoing warfarin treatment.

To explain the discrepancies in INR observed in this patient, one would expect some secretion of the Gly244Arg variant. As mentioned, the *F10*:p.Gly244Arg variant has been shown to cause type 1 deficiency in homozygous and compound heterozygous patients due to destabilization of the disulfide bond and intracellular degradation [5]. Unfortunately, we did not have access to a chromogenic FX assay to investigate whether the Gly244Arg variant was secreted. Possible causes that could explain the secretion of *FX*:p.Gly244Arg in our patient, and thus its interaction with thromboplastin, include decreased gamma-carboxylation due to warfarin treatment, resulting in a change in posttranslational modification and secretion of the dysfunctional variant, or that the heterozygous state allowed for the protein to be carried in secretory vesicles along with the correctly processed wild-type protein. However, this remains speculative.

Unfortunately, we do not have any spontaneous (ie, without anticoagulant treatment) POCT INR using human thromboplastin. However, when comparing INR measurements during vitamin K antagonist treatment (Figure), we expect this to be normal. Nonetheless, the importance of this case report lies not in the exact value but in the difference between the INR measurements obtained with the different thromboplastins.

## 4 Conclusion

In conclusion, the current case report indicates that *F10*:p.Gly244Arg heterozygosity is associated with a more pronounced increase in PT when using an assay based on rabbit thromboplastin compared with an assay based on human thromboplastin. In vitamin K antagonist-treated patients, this phenomenon could lead to falsely elevated INR measurements using thromboplastin from nonhuman sources. This, in turn, could lead to undertreatment with a vitamin K antagonist if the therapeutic range is incorrectly lowered due to differences in interactions with thromboplastin, resulting in an increased risk of thrombosis.

A significant discrepancy between prothrombin and INR measurements should lead the treating clinician to consider the possibility of genetic variants in relevant coagulation factors (eg, FII, FVII, and FX when using Owren PT-based INR), especially when a POCT INR based on human thromboplastin measurements yields a lower PT/INR than an assay based on, eg, rabbit thromboplastin.
